# Next-Generation Cardiac Magnetic Resonance Imaging Techniques for Characterization of Myocardial Disease

**DOI:** 10.1007/s11936-024-01044-4

**Published:** 2024-08-09

**Authors:** Julia Simkowski, Brendan Eck, W. H. Wilson Tang, Christopher Nguyen, Deborah H. Kwon

**Affiliations:** 1 Heart, Vascular, and Thoracic Institute, Cleveland Clinic, 9500 Euclid Avenue, Cleveland, OH 44195, USA; 2 Diagnostic Services, Cleveland Clinic, Cleveland, OH, USA; 3 Biomedical Engineering, Lerner Research Institute, Cleveland Clinic, 9500 Euclid Avenue, Cleveland, OH 44195, USA; 4 Cardiovascular Innovation Research Center, Heart, Vascular, and Thoracic Institute, Cleveland Clinic, 9500 Euclid Avenue, Cleveland, OH 44195, USA

**Keywords:** Cardiac MRI, Quantitative Parametric Mapping, Magnetic Resonance Fingerprinting, Diffusion Tensor Imaging, Artificial Intelligence

## Abstract

**Purpose of the Review:**

Many novel cardiac magnetic resonance imaging (cMR) techniques have been developed for diagnosis, risk stratification, and monitoring of myocardial disease. The field is changing rapidly with advances in imaging technology. The purpose of this review is to give an update on next-generation cMR techniques with promising developments for clinical translation in the last two years, and to outline clinical applications.

**Recent Findings:**

There has been increasing widespread clinical adoption of T1/T2 mapping into standard of care clinical practice. Development of auto segmentation has enabled clinical integration, with potential applications to minimize the use of contrast. Advances in diffusion tensor imaging, multiparametric mapping with cardiac MRI fingerprinting, automated quantitative perfusion mapping, metabolic imaging, elastography, and 4D flow are advancing the ability of cMR to provide further quantitative characterization to enable deep myocardial disease phenotyping. Together these advanced imaging features further augment the ability of cMR to contribute to novel disease characterization and may provide an important platform for personalized medicine.

**Summary:**

Next-generation cMR techniques provide unique quantitative imaging features that can enable the identification of imaging biomarkers that may further refine disease classification and risk prediction. However, widespread clinical application continues to be limited by ground truth validation, reproducibility of the techniques across vendor platforms, increased scan time, and lack of widespread availability of advanced cardiac MRI physicists and expert readers. However, these techniques show great promise in minimizing the need for invasive testing, may elucidate novel pathophysiology, and may provide the ability for more accurate diagnosis of myocardial disease.

## Introduction

Recently, significant advances in treatment for various cardiomyopathies, including cardiac amyloidosis, genetic cardiomyopathies, and heart failure with preserved ejection fraction, heighten the need for early and accurate diagnosis, as well as robust risk prediction, to enable early treatment and improved outcomes [1]. cMR technology has been developed to perform non-invasive assessment of cardiac function, structure, hemodynamics, tissue characterization, perfusion, and viability amongst other indications. However, a typical cMR is performed with gadolinium-based contrast, and requires multiple breath holds, EKG gating, and prolonged image acquisition time, thus limiting patient tolerance and image quality, especially in patients with decompensated heart failure. Additionally, cardiac devices often result in significant susceptibility artifact with certain cMR imaging techniques, and advanced technical expertise is often required to fully optimize CMR protocols for diagnostic imaging, These limitations have led to significant advances in CMR image acquisition and processing, some of which have started to be adopted and translated into the clinical arena. This review article will outline novel techniques that have been published over the last 1–2 years with the purpose of updating the general, imaging, and heart failure cardiologist on the basic physics principles and clinical applications of these new techniques.

## Advances in Current Standard MRI Techniques

The essential components of a standard cMR acquisition include T1 and T2 weighted sequences, cine functional analysis, perfusion imaging, and late gadolinium enhancement sequences. Additionally, cMR enables comprehensive assessment of relevant cardiomyopathic associations, such as wall thickness, secondary valve regurgitation, resultant distortion of valve/papillary muscle architecture, and microvascular dysfunction. The following section serves to give a brief overview of these imaging sequences to provide a backdrop for how recent developments in cMR expand and augment current cMR capabilities.

### Late Gadolinium Enhancement (LGE)

LGE classically differentiates replacement fibrosis from healthy myocardium and has become the gold standard for tissue characterization and categorizing cardiomyopathy based on LGE patterns [2]). Furthermore, numerous studies have demonstrated the powerful prognostic utility of LGE. Therefore, LGE is a key core component of standard CMR protocols. However, limitations with spatial resolution and motion artifacts that obscure definitive diagnosis of myocardial disease are not infrequent. Furthermore, heterogeneity of LGE characterization in regards to signal intensity, extent of LGE, number of discrete non-contiguous areas of LGE, and LGE location can impact overall interpretation and inter-observer variability. High resolution 3D LGE enables the ability to identify much smaller and focal areas of LGE that may not otherwise be able to be discerned with standard segmented LGE, which can result in reclassification of disease [3]. Recently, a free breathing technique with respiratory motion correction (MOCO) has significantly improved image quality, particularly in patients who have difficulty performing consistent and good quality breath holds [4].

### CINE Imaging

CINE imaging is a standard part of cMR image acquisition and is typically acquired using steady state free precession (SSFP) techniques. CINE imaging provides valuable information about wall motion, ventricular volumes, and ventricular mass. It can also be used to identify valvular disease and morphologic abnormalities. Novel techniques using cine imaging include black blood cine and magnetic resonance fingerprinting (MRF) cine have demonstrated feasibility of acquiring tissue characterization simultaneously with wall motion assessment [4, 5]. Additionally, 3D whole heart cine can be acquired to enable a fully contiguous imaging data set with full flexibility to modify imaging planes during post-processing [6, 7]. Deep learning has also been applied to cine imaging to enhance de-noising, and significantly accelerate image acquisition and image reconstruction. Such developments may significantly improve patient tolerability, image quality, scan time, and diagnostic assessment.

### Perfusion and Fully Automated Perfusion

Stress cMR has several strengths over nuclear stress testing due to its superior spatial resolution, and unique tissue characterization, resulting in improved sensitivity [8]. Severe or hemodynamically significant coronary artery stenosis is identified by hypoperfusion and resultant decreased signal in corresponding myocardial segments.

Stress perfusion CMR using adenosine or regadenoson can also provide myocardial perfusion reserve (MPR), or coronary flow reserve, and is defined as the ratio of myocardial blood flow at rest and stress. Measurement of MPR with cMR is currently limited by time intensive post-processing. Novel methods of fully automated perfusion mapping could close this gap, but is not yet FDA approved. Some examples of fully automated cMR include the assessment of epicardial and microvascular disease. Kotecha et al. used a novel automated inline pixel by pixel perfusion mapping technique and demonstrated significant ability to both identify epicardial and microvascular disease, and in the absence of a regional perfusion defect, it could differentiate triple vessel disease from microvascular disease [9].

Stress cMR now has emerged with Class I indications for the evaluation of patients with cardiac chest pain, and is the only imaging modality that can provide unique advantages for evaluation of patients with MINOCA, microvascular disease, pericarditis, and myocarditis [10]. Stress cMR has been used to develop a myocardial perfusion reserve index for non-invasive diagnosis of microvascular disease. The myocardial perfusion reserve index was associated with increased MACE in patients with chest pain and non-obstructive coronaries (predominately in HFpEF patients) [11]. Microvascular disease has also shown to be highly prevalent in NICM patients as diagnosed by stress cMR [12]. However, more common use of stress cMR is limited due to limited access, decreased accuracy in patients with significant arrhythmias, patient intolerance due to claustrophobia, device related artifacts.

Novel techniques for cMR using exercise stress testing are emerging. Widespread clinical adoption of exercise cMR was limited in the past by availability of compatible equipment, limited ECG monitoring of ST segment changes during exercise, and significant motion artifacts due to elevated heart and respiratory rates during exercise. However, MR compatible treadmill and supine cycle ergometers have been developed with prior studies demonstrating clinical feasibility [13, 14].

Advanced techniques have recently been developed to leveraging deep learning to improve image quality, making more widespread adoption more feasible [15]. In regards to technical issues with acquiring cine imaging immediately after exercise, free breathing real time cine images can be acquired to evaluate gross wall motion abnormalities. The EMPIRE trial utilized this technique and included patients with angina and at least one cardiovascular disease risk factor who were referred for PCI after undergoing exercise stress cMR performed with bicycle ergonometer. Multi-parametric data offered the strongest c-statistic for discrimination of FFR abnormal to FFR normal patients (including LGE, stress inducible regional WMA, perfusion defects, and peak exercise cardiac index percentile-rank) [16].

### Quantitative Parametric Mapping

#### T1 Mapping and Extracellular Volume Fraction Quantification (ECV)

More widespread clinical adoption of T1 mapping and ECV measurements have been adopted to provide quantitative assessment of myocardial interstitial fibrosis and deposition [17]. T1 quantifies the course of recovery of longitudinal magnetization with recovery time reflecting the property of the tissue [18]. Pre and post contrast T1 measurements are used to calculate ECV, and normalized to the hematocrit. The ECV (or the interstitium) expands with infiltrative disease, extracellular edema, or myocardial fibrosis. Widespread clinical adoption has been increasing, but currently there is no standard accepted global reference range. However, elevated ECV (due to increased fibrosis or collagen deposition) has been associated with all-cause mortality in non-ischemic cardiomyopathy patients, and associated with hospitalization and cardiac death in heart failure with preserved ejection fraction (HFpEF) [19]. Other novel techniques include synthetic ECV (quantification of ECV without a blood sample for hematocrit) and wideband T1 mapping (technique to minimize artifact associated with cardiac devices such as ICD to enable more accurate identification of scar and myocardial fibrosis) [20, 21].

#### T2 Mapping

T2 relaxation times represents transverse magnetic relaxation of tissue and is used to visualize edema (T2 relaxation time is lengthened when edema is present due to interstitial free water). T2* is used to visualize iron deposition. Clinical use and evidence for T1, T2, T2*, and ECV as of 2017 is summarized in the consensus statement from the Society of Cardiovascular Magnetic Resonance [17].

## Next-Generation Techniques to Know for the General Cardiologist (Technique, Implications, Selection Criteria)

### Diffusion Tensor Imaging (DTI)

DTI provides the ability to visualize and measure alterations in the microstructure of the myocardium (directionality and orientation of micro fibers) in vivo [22, 23] DTI is based on the principle that in myocardial tissue, the myocytes and connective tissues act as barriers to diffusion, scattering the path of diffusion and making it anisotropic. This anisotropy is captured by cMR by quantifying the loss of signal due to tissue barriers. Diffusion gradients are generally applied in at least 6 directions and then quantified in the form of a vector with 3D magnitude and direction [24]. The STEAM technique (stimulated echo acquisition mode) was the first technique described for in vivo diffusion imaging of the myocardium [25]. It measures diffusion across an RR interval over 2 regular cardiac cycles with a breath hold which limits its clinical applicability. Early work in DTI has been focused on better characterizing the histology of the myocardium and understanding the microstructure changes responsible for radial wall thickening in systole, including fiber orientation throughout the myocardial wall, and sheetlet rearrangement [22, 26]. Additional clinical investigations have looked at post myocardial infarction, hypertrophic cardiomyopathy, amyloidosis amongst others [27, 28].

Eder, Nguyen et al studied the distribution of CITED4 expression and the local microstructure tissue helicity mouse cohorts using DTI-MRI. They found that they correlate in sedentary and exercised mice, suggesting that CITED4 regulation of cardiac remodeling impacts exercise-induced regional remodeling of the heart [29]. Future directions of DTI might include expanding the role of DTI to help predict which patients with congenital or ischemic heart disease are at risk of developing heart failure.

However, wide applicability continues to be limited by cardiac and respiratory motion, variations in image acquisition, long scan times, limited image resolution, and the unknown impact of strain. The data produced is averaged across a voxel, however that voxel contains millions of myocytes and sheetlets that may not be uniformly aligned, suggesting that histologic information is being missed by averaging [30]. The influence of myocardial strain on the results obtained with diffusion tensor imaging is debated [31].

To address these limitations, developing techniques within DTI are aimed to enable better clinical adoption. M2-SE (second order spin echo) is a motion compensation technique that is commonly used. This DTI technique provides good signal to noise ratio and a free breathing technique can be applied. Nguyen et al published a free breathing M2-MT-MOCO technique to allow DTI with free breathing for patients with symptomatic HF [32].

### Magnetic Resonance Fingerprinting (MRF) and Multi-Parametric Mapping

MRF is a multiparametric cMR technique that has been developed to quantify and characterize the tissue of interest using different tissue contrasts in a single acquisition. The primary use to date has been to perform simultaneous T1 and T2 mapping in a single breath hold, which can shorten long scan times and provide inherent co-registration of T1 and T2 maps without additional post-processing. The goal of MRF and multi parametric acquisition is to take advantage of the redundancy in cMR image acquisition and extract a variety of information from one scan sequence. While imaging, sequence parameters are varied throughout image acquisition (e.g. flip angle, repetition time, magnetization preparation pulses) to yield unique signals for each combination of T1, T2, and potentially more, tissue property values. These signals represent a tissue’s ‘fingerprint’ that can be extracted in a voxel-wise fashion and compared/matched to a patient specific dictionary of fingerprints determined based on each scan’s unique, precise timings that include the cardiac rhythm recorded at the time of acquisition. This allows scans to be more efficient and improve signal to noise ratio and motion artifacts [23].

Multi-tasking: Multi-tasking is a well described free breathing multi parametric sequence without need for EKG gating [33]. T1 and T2 maps are generated from an under sampled image acquisition using low rank tensor or other reconstruction methods. Because the entire T1 and T2 recovery period was acquired, various cine stacks can be constructed (dark-blood, bright-blood, T1 or T2 weighted). Fat, scar, edema and myocardium can be studied if recovery times are known.

MRF still exists primarily in a research space, is the body of literature using cardiac MRF is growing with continued development for more widespread clinical implementation. Prior studies have demonstrated robust agreement with conventional T1, T2, and ECV in different cardiac disease cohorts, with improved image quality and reproducibility compared to conventional methods [34].

MRF also enables the ability for additional quantitative assessments. The Dixon method of MRF was developed to acquire T1, T2, and M0 (fat fraction) in a single breath hold [35], using radial acquisition and HD-PROST reconstruction and was validated in a cohort of healthy subjects [36]. Cao et al also developed a free breathing technique using a multi-tasking and low rank tensor method to simultaneously acquire T1, T2, T2* and fat fraction mapping in phantom patients and healthy controls [37]. There were some differences between values acquired from the novel vs traditional methods however with acceptable image quality and repeatability. Lima da Cruz et al also refined their method to include T2* with improved precision compared to standard methods [38]. Myocardial T1-rho mapping has emerged as a possible biomarker to quantify myocardial injury without the need for contrast administration however is still in the early stages of development [39].

MRF provide hundreds of measurements with the ability to enable additional tissue characterization beyond T1 and T2. Conventional T1 and T2 mapping likely provides incomplete tissue characterization as it assumes that all tissue with a single voxel have the same T1 and T2 relaxation time.; however, a single voxel represents millions of myocytes, mostly likely with varying T1/T2 properties. However, MRF enable novel tissue characterization while still providing T1 and T2 assesesment as illustrated by preliminary work in cardiac amyloidosis has shown that MRF has the potential to provide better tissue characterization than either T1 or T2 alone [40].

### CMR Elastography is a Non-Invasive Measure of Myocardial Stiffness

Mechanical motion from an external driver induces shear waves in the tissue of interest. The displacement of the shear waves get encoded in a phase contrast MRI sequence. Motion encoding gradients are synchronized with external motion, and processed by a mathematical inversion algorithm to produce stiffness maps [41]. A gated cine sequence is commonly used to acquire images. A variety of image acquisition techniques have been proposed [42]. MR Elastography generated stiffness-volume loops have been compared to PV loops in animal models, with good correlation. Contractility and stiffness post infarction have also been studied in animal models. MRE using human subjects remains in the research space, with ongoing investigations including use for MRE in staging liver fibrosis and characterizing tumors. Cardiac application has so far been limited by need for prolonged breath holds and EKG gating, high stiffness myocardium with relatively smaller ventricular walls (compared to skeletal muscle for example), anisotropic mechanical property of tissue, and the bulky external mechanical wave source needed. Novel research has developed a technique to use the mechanical shear waves generated by aortic valve closure rather than an external source [43] which has showed significantly different values of stiffness and acceptable inter observer variability in small populations of cardiac patients with increased wall thickness.

### 4D Flow (Time-Resolved Three-Dimensional Phase Contrast cMR)

The novel technique of 4D flow involves acquiring data about velocity of flow in 3 directions in order to calculate volumetric data over time (3 directions plus time = 4D) [44]. It can be done using a free breathing technique with respiratory and ECG gating. 4D flow has been used to study flow in major vessels, and with developing technology, is more commonly used for intracardiac flow and imaging of anatomic structures. It has been validated against echo and 2D flow cMR [45]. Scan acquisition times are lengthy and can be shortened by various techniques (parallel imaging, compressed sensing, non-Cartesian trajectory techniques) although at the expense of spatial and temporal resolution. The maximum expected velocity must be estimated and if too low, aliasing artifacts can result. The most time-consuming portion of 4D flow is post processing due to large amounts of data acquired, which is a potential window for machine learning to streamline processing. One study using a swine model with 9 subjects showed that cardiac output correlated well between 3D flow and directly measured flows using an invasive probe placed around the aorta (Stam et al.). 4D flow is being applied across a variety of cardiac conditions including congenital heart disease, valvular heart disease, and quantification of kinetic energy within the heart. It has been applied to assess flow patterns in patients with different phenotypes of bicuspid aortic valves and the subsequent impact on shear stress on the aortic wall [46].

### MR Spectroscopy (MRS) and Chemical Exchange Saturation Transfer (CEST)

MR spectroscopy is based on the concept that in various cardiomyopathies, the levels and distribution of metabolites in the cardiac tissue changes and can be imaged. The technique quantifies metabolites non-invasively through MR active cardiac nuclei (protons 1H, phosphorous 31P, and carbon 13C), knowing their nuclei resonate at a different frequency than the hydrogen in water (ie typical MR imaging). Most commonly, 1H MRS is used to identify total creatinine, taurine, lactate, alanine, deoxymyoglobin, carnitine, and fatty acids. 1H MRS has been used in the past to characterize myocardial infarction and ischemia, heart transplant, and gestational cardiomyopathy amongst others [47]. It more successfully characterizes disease state with large changes in metabolite levels.

MRS using 31P and 13C are used to assess cardiac metabolism via the creatinine kinase system. It can assess PCr/ATP ratio, intracellular pH, CK flux, [PCr], and [ATP] and give information about in vivo cardiac metabolism not feasibly accessible from 1H MRS. One study used hyperpolarized 13c MRS to evaluate the hearts of 13 people with DMII compared to 12 healthy controls. Myocardial energetics (31P) and lipid content (1H) were imaged fasting. 1–23C pyruvate was used to image activity of pyruvate dehydrogenase and showed increased carbohydrate metabolism after glucose challenge with impaired response in diabetics. The results showed abnormal myocardial energetics, lipid content, and diastolic dysfunction in diabetic patients [48].

Most work on proton MR spectroscopy is done at clinical strength machines (ie 1.5T) however there is limited sensitivity (small concentration of the metabolite, overlap of the signal from different nuclei) and this requires long scan times. Higher field strength (3T and 7T) introduces chemical shift displacement error and magnetic fields inhomogeneity resulting in localization error which can be mitigated by advanced single voxel spectroscopy (SVS) protocols including SPECIAL and sLASER pulse sequences [49].

Chemical exchange saturation transfer (CEST) is a new technique that detects the change in concentration of metabolites that are too low to be seen in standard MR contrast or with MR spectroscopy. The technique creates imaging contrast between water and the metabolite of interest. More advanced physics and imaging techniques are required to create clinically useful images [50]. As CEST comes closer to clinical application, deep learning approaches will be beneficial in this low signal-noise ratio technique [51]. One study used MRF to conduct simulations, each varying different imaging parameters and examined the effect on the CEST signal to optimize sensitivity, reduce scan time, and lower required saturation power [52]. However, most CEST imaging has been limited to outside of the heart and significant technical challenges remain to translate those techniques to cMR.

### Technological Advances

- In addition to MRI acquisition and post processing advanced, the technology itself and how we use it is rapidly changing. Some advances include advances in soft robotics and 3D printing, which have been used to create models of cardiac pathology that can be then used to advance our knowledge of hemodynamics. For example, Nguyen, Rosalia et al. created a soft robotic aortic sleeve with the assistance of computational modeling, intended to mimic aortic stenosis that including bicommissural and unicommissural defects. 4D flow was acquired and analyzed via cMR to better understand patient hemodynamics. This technology could improve personalized treatment of aortic stenosis [53].
- Advances in cMR technology will also further the amount of detail and microstructure that can be imaged using cMR. Recently there has been approval for clinical use for 7 T scanners from several vendors. High field systems have the potential advantage of improved tissue contrast and shorter scan times, however technical limitations have limited clinical applicability thus far [54].
- Recent machines developed include the Magnetom Cima.X (3 T field) and Magnetom Terra.X (7 T field), both of which offer increased speed and gradient speed than prior models (Siemens Healthcare©). However there has been renewed interest in low field systems due to reduced cost and potential to improve accessibility, and one study showed comparative results between a 0.55 T system compared to a conventional 1.5 T system [55].

## Important Derivatives of advanced cMR

### Radiomics

Traditionally, medical images have been interpreted based on visual interpretation alone. Radiomics represents a new frontier of cardiovascular imaging, in which the image represents a set of data to be extracted, quantified and mined, and analyzed using statistical techniques. The goal is to turn the mineable data into new imaging biomarkers that support clinical decision making [56]. Radiomics has been used to study cardiomyopathies in which application of traditional imaging is limited, and has been part of the drive to improve precision medicine. Wide applicability is limited by differences in image acquisition and reconstruction, and the amount of data needed to detect a signal. When mining the data for patterns, machine learning algorithms have been applied including supervised and non-supervised approaches (neural networks, support vector machines, or Bayesian networks). This working is increasingly being applied to phenotype cardiomyopathies.

Texture Analysis of native T1 mapping is the analysis of the variability of pixel intensity and the relationship between neighboring pixels in a region or tissue of interest. Texture analysis has been studied in multiple cardiac pathologies including dilated cardiomyopathy, acute infarction, hypertensive heart disease, HCM, myocarditis, and HFpEf related to ESRD [57, 58].

Radiogenomics is a new development in the field of cMR which integrates imaging data (radiomics) with molecular markers. It combines radiomics of certain genomic phenotypes to build prediction models, guide therapy, and assess outcomes. Integrated databases are key to discovering meaningful information [59]. Thus far there has been little investigation to the use of radiogenomics in cardiac patients.

### Deep Learning and Artificial Intelligence

Machine learning is defined as computer algorithms that are programmed to learn through experience, permitting relatively efficient analysis of large data sets. A predictive model is built based on a training data set without being coded to specifically make that decision; more specifically, such models typically have a large number of parameters to be optimized for a target outcome (loss function) given a training dataset. Deep learning is broadly defined as a subset of machine learning where complex artificial neural networks are used to identify abstract representations of data that are then used to accomplish a task [60]. The benefit of deep learning over traditional logistic regressions is that it can describe complex and non-linear relationships. However, this makes understanding both the clinical applicability of the outcome and where the model fails difficult. The power of a deep learning network is dependent on having very large sample sizes and is generally split into a training cohort and the testing cohort [61]. Commonly used algorithms include neural networks, support vector machines, decision trees, and Bayesian methods. As in radiomics, lack of publicly available data remains a limitation of widespread applicability.

Previous articles of this journal [60, 61] have reviewed machine learning for cardiac risk prediction and cardiomyopathy in detail. This review will therefore focus on recent advances in machine learning and artificial intelligence as applied to cardiac MRI that help reduce computational demand, improve image quality, and extract more information from cMR images. For cardiac MRI, the input data for deep learning algorithms are generally from the scanner (signal evolutions, k-space data, reconstructed images) [62]. One example of machine learning is its application to MRF. Bloch equations are used to simulate an MRF sequence and thus generate dictionaries used in multi parametric reconstruction, but these dictionaries grow in size depending on the number of tissue properties of interest. Machine learning has been employed to substitute full Bloch equation simulations and shorten processing time for creating dictionaries, expanding the capabilities of MRF.

Machine learning is also being studied to improve image de noising and cMR post processing [63, 64].

To better understand how machine learning can advance our knowledge of cardiomyopathy, it is helpful to review the body of work that has been done for patients with hypertrophic cardiomyopathy. For example, Zhang et al proposed a novel machine learning technique to create images similar to LGE without need for contrast and tested their model on a cohort of HCM patients. This technology called virtual native enhancement is a deep learning model that uses multiple streams of convolutional neural networks with input of native T1 maps and vine images and output of an LGE like image. The images produced by this method showed good agreement with distribution and quantification of LGE lesions with higher image quality and faster scan times [65]. The same group applied this virtual native enhancement technology to patients with myocardial scar post infarction also with good agreement with standard LGE images [66]. Fahmy et al studied a radiomics model, a deep learning model, and a combined model of the two to assess for patients with HCM without scar, it identified 28% of patients without scar in the external data set, but clinical utility is limited based on these results [67]. The same group studied a deep learning model that combines LGE and cine images vs a model with just LGE images for LGE scar quantification, both of which had good agreement with manual quantification of scar burden, suggesting an automated model could be clinically useful [68]. Farrag et al studied the application of CNN (U net, densely connected neural networks, and Attention nets) to the automated segmentation of native and contrast enhanced T1 maps, with U-Net showing the best results in automated myocardial segmentation of native T1, contrast enhanced T1, and cine images. A CINE based method in which CINE images are contoured automatically using U-Net and those contours are applied to the corresponding regions of T1 maps outperformed U-Net in segmentation of contrast enhanced T1 maps, especially in ischemic cardiomyopathy patients [69].

## Cardiomyopathies of Interest

Table 1 highlights how the novel techniques described above have been applied to our understanding of a variety of cardiomyopathies.

### Precision Medicine and Personalized Medicine

Precision medicine and personalized medicine are concepts within healthcare that diagnosis, decision making, and treatment should be individualized to the patient. This requires multiple information inputs, including but not limited to clinical tests, big data and machine learning, imaging, and genomics. With advances in cMR, focus has turned to use radiomics and deep learning to analyze and extract rich data and information from traditional cMR sequences that are commonly performed, and develop disease phenotypes, with the end goal of early and accurate diagnosis to guide targeted treatment, enhance risk prediction, and improve outcomes [83].

Phenomapping is a component of individualized medicine (including risk prediction and prevention) that requires machine learning analysis. Cluster analysis with unsupervised algorithms can reveal previously unrecognized phenotyping groups. One study by Anthony et al performed cluster analysis on a cohort of ICM patients with cMR looking for gender specific predictors of survival. Four unique phenotypes were identified and differed between women and men suggesting that cluster analysis and cMR could be a powerful tool in advancing individualized medicine and risk stratifying patients [84]. An additional study used an unsupervised machine learning to examine patients with newly diagnosed CAD who had data from coronary CTA and vasodilator stress cMR in addition to common clinical data. This was available to identify three unique phenogroups (elderly patients with few traditional risk factors, women with metabolic syndrome, calcified plaque on CCTA and preserved LVEF, and younger male smokers with proximal non-calcified plaques on CCTA myocardial scar and reduced LVEF) with one group having higher MACE, CV mortality, and all-cause mortality [85]. These two studies had many limitations that limit clinical applicability, however they demonstrate that as cMR continues to advance with corresponding advances in radiomics and machine learning, there is a great opportunity to advance personalized medicine in treatment of cardiomyopathy.

In addition, a recent study by Kwon et al applied an unsupervised phenomapping algorithm to a cohort of patients with mitral regurgitation and ischemic cardiomyopathy (a significant percentage of whom underwent cardiac surgery). Using latent class analysis with a pre-defined set of cMR and clinical variables they were able to identify three distinct clusters of patients with differential survival benefit after revascularization and mitral valve repair. This highlights a potential novel approach to careful and personalized patient selection for cardiac surgery [86].

## Figures and Tables

**Table 1:** Novel cMR techniques for the Evaluation of Various Cardiomyopathies

Technique	Picture	Advance	Example	
Automated Perfusion	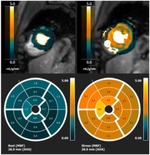	More rapid post processing promises to expand the clinical utility of perfusion cMR.	ICM: Hsu et al performed traditional perfusion imaging on 80 pts with known or suspected CAD and 17 controls, using manual methods and a novel fully automated method with good correlation between the two. The fully automated method was able to detect areas of the myocardium with lower stress MBF due to ischemic heart disease. [70]	
Exercise cMR	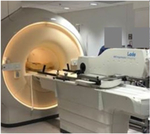	Non invasive diagnosis of HFpEF in the exercising patient. Expanded application of stress cMR.	HFpEF: Backhaus et al studied 34 pts with shortness of breath and suspected HFpEF with echocardiography, exercise RHC, standard cMR (with strain) and real time exercise cMR (supine ergometer). LA dysfunction outperformed LV in ability to diagnose HFpEf. HFpEF patients had significantly lower LA strain compared with patients with non-cardiac dyspnea at exercise suggesting possible utility of stress cMR for early diagnosis of HfpEf [71].	DCM: Le et al examined a cohort of 60 DCM patients vs 100 controls with a stress cMR supine ergometer). Cardiac index at peak exercise in patients with suspected early pathologic dilated cardiomyopathy was lower than healthy patients with remodeling due to frequent activity. Only pts with DCM experienced adverse events (limited by lack of long term follow up) [72]
CEST and Spectroscopy	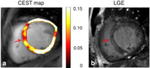	A more nuanced understanding or the impact of various cardiomyopathies on cardiac metabolism.	ICM: One study induced cardiac MR in pigs and did standard MR imaging including LGE 3 days after MI to characterize the infarct region. Creatine weighted CEST was performed (exchange of NH2 protons in creatine with water protons) to assess impact of MI on creatine kinase phosphagen system. The results showed decrease creatine at day 3 with and significantly elevated creatine on day 14 post MI suggesting functional recovery of the myocardium [73].	ICM: 1H and 31P MRS have been used in the past to demonstrate that in infarcted tissue, [Cr], PCr/ATP ratio, [PCr], and [ATP] are significantly reduced. It offered in vivo evidence that cardiac metabolism is decreased in at risk and non-viable tissue [47].
			HCM: Neisius et al studied texture signatures in 188 patients with suspected or known HCM. LGE images were location matched with native T1 maps, and a training, internal validation and cohorts identified five texture signatures could discriminate between LGE+ and LGE negative T1 maps with a c statistic of 0.74 in the independent test, suggesting gadolinium could have been avoided in that patient group. [74]	HFpEF: In 119 pts with ESRD, adding significant texture analysis features extracted from native T1 maps in HFpEF pts to pts with ESRD without HFpEFF, to a predictive model including age and LV GLS, had good sensitivity and specificity for diagnosis of HFpEF. [57].
Radiomics	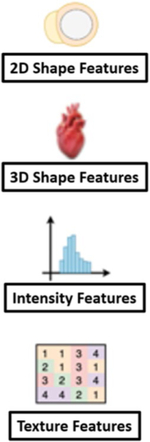	Novel biomarker to identify region of interest. For example, texture analysis uses more common cMR acquisitions (ie native T1) to extract richer information for diagnosis and management	HCM: Pu et al studied 273 patients with HCM with goal of creating a model to identify patients at risk of fibrosis without exposure to contras. Imaging features predictive of HCM were combined in an integrated model with radiomic features and this integrated model was assessed on a test cohort. Radiomic features enhanced the predictive power of the imaging features alone model in identifying LGE positive patients [75]	HCM: Antonopoulos et al studied 149 patients (healthy control, LVH, HCM, and cardiac amyloidosis) and extracted 850 T1 radiomic features. A T1 map of a single basal slice was used for texture analysis. A machine learning algorithm for feature selection was applied. A model of 6 radiomic features was found to have better discriminatory ability between phenotypes of hypertrophied muscle compared to native T1 values suggesting this proof of concept study’s potential clinical application [76].
Machine Learning	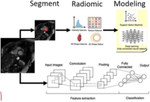	Increasing the speed of post processing of current and developing cMR techniques.	Brahim et al developed a 3D deep learning model for automated segmentation of the myocardium, infracted tissue, and microvascular-obstructed tissues from LGE-MR images using the inclusion and classification of prior information U-NET (ICPIU-NET), with good performance of the model. It is currently limited to research applications [77].	DCM: Li et al created an automated model to first contour and segment a patient’s cardiac MRI data, perform strain analysis, and predict patients with dilated cardiomyopathy using machine learning. The results were comparable to deep learning models requiring larger data sets [78].
MRF [79]*	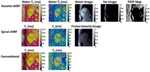	Faster acquisition of multiple cMR tissue properties (e.g. T1, T2, PDFF) with fewer breath holds than conventional mapping methods; Can provide cine imaging in the same scan as tissue property mapping; Opportunity for synthetic LGE	DCM: 9 patients with DCM underwent single breath hold acquisition with contrast enhancement. ECV Native T1 measured with MRF and MOLLI were higher in DCM pts than controls. Native T1 and T2 values were lower than traditional in DCM and control pts [80]	
DTI	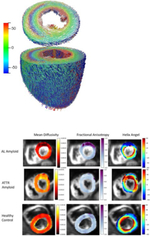	Improved understanding of cardiac microstructure and how it varies within and between cardiomyopathies and how this aids in non-invasive diagnosis	HCM: Das et al studied twenty patients with HCM. In segments considered normal by traditional imaging (normal wall thickness, normal perfusion, no LGE), HCM patients had significantly increased mean diffusivity, E2A, and decreased fractional ansiotropy with more abnormal values in the subendocardium. 42% of segments with normal wall thickness had abnormally low perfusion. Elevated E2A suggets hypercontracted formation of myocardial sheetlets during systole with impaired relaxation during diastole, more so in hypertrophied segments. This suggests DTI could be a powerful tool to identify early microstructural changes and abnormal microvascular perfusion in HCM patients. [81]	HCM: Das et al performed a similar study using DTI to investigate differences in microstructure of HCM vs normal vs athletic hearts. HCM patients had elevated MD, E2A, and lower FA then both athletes and volunteers, and significnatly elevated ECV. This study demonstrated a direct correlation between MD and LGE values in patients with HCM. The results suggest that the microstructure of hypertrophy in HCM is due to increased fibrosis and myocyte disarray compared to an athletic heart which thickens via hypertrophy [82].

* The MRF pictures are from *Front Cardiovasc Med*, 2022. 9: p. 977603; https://doi.org/10.3389/fcvm.2022.977603; Creative Commons user license https://creativecommons.org/licenses/by/4.0/[70]

## Opinion Statement

As therapeutic options for various cardiomyopathies are expanding, the need for advanced imaging to provide early and accurate diagnosis as well as robust risk prediction is becoming more critical. Novel techniques are pushing the boundaries of the field and implementation of machine learning and artificial intelligence applications are entering into clinical practice These continuing advancements continue to strengthen the unique capabilities for cMR to provide precise and accurate assessment of structural abnormalities, cardiac function, and myocardial tissue characterization. Advanced cMR continues to provide innovative opportunities for enhancing modern management of cardiac disease.

## Future Directions

T1 and T2 mapping have advanced the field of cMR for management of cardiomyopathy and have started the effort to reduce need for gadolinium-based contrast, however traditional methods continue to be limited by need for EKG triggering, respiratory motion, and lengthy scan times. Radiomics, machine learning, and MRF have taken T1 maps, LGE, and ECV and extracted richer data, to build accurate diagnostic models in testing cohorts. MR spectroscopy and CEST, strain analysis, exercise stress cMR, and 4D flow have revolutionized evaluation of the diseased myocardium by cMR. Diffusion Tensor Imaging has shown great promise in establishing a technique for non-invasive biopsy and tissue characterization, but still has multiple limitations before it will be widely clinically available. Widespread clinical application is currently limited by reproducibility of these novel proof of concept techniques. Given recent advanced and ongoing research, the addition of biomarkers and genomics, cMR will be a powerful tool in correctly diagnosing, prognosticating, and monitoring cardiomyopathy phenotypes.
